# The Predictive Power of Positive Memory Therapy (Nostalgia) in Enhancing the Sense of Belonging and Tranquility among the Elderly

**DOI:** 10.1192/j.eurpsy.2026.11595

**Published:** 2026-07-31

**Authors:** D. M. Al Majali, D. H. Dudin

**Affiliations:** 1 Education, Dhofar University, Dhofar, Oman; 2 Education, Hebron University, Hebron, West Bank and Gaza

## Abstract

**Introduction:**

Aging involves gradual changes in physical, cognitive, and emotional functions, often leading to social isolation, anxiety, and reduced life satisfaction. Positive memory therapy, or nostalgia, has emerged as a non-pharmacological intervention that reconnects older adults with meaningful past experiences, enhancing psychological well-being, social engagement, and emotional stability. Previous studies highlight nostalgia’s role in improving mood, social interaction, and sense of security.

**Objectives:**

This study aimed to

1. assess the level of nostalgia among older adults in a Bethlehem nursing home,
2. measure their sense of belonging and tranquility,
3. examine nostalgia’s predictive capacity in enhancing belongingness and tranquility,
4. explore differences by gender and length of stay.

**Methods:**

A descriptive correlational design was used. The population included 70 older adults, with a random sample of 40 participants. Data were collected using a validated and reliable 46-item questionnaire covering nostalgia, belongingness, and tranquility. Responses were scored on a five-point Likert scale. SPSS was used for descriptive statistics, t-tests, and simple regression analyses.

**Results:**

Participants exhibited high levels of nostalgia (M = 4.56, SD = 0.31) and belongingness (M = 4.65, SD = 0.22). Nostalgia significantly predicted belongingness (R² = 0.116, p = 0.032) and tranquility (R² = 0.255, p < 0.001). No significant differences were observed based on gender or length of stay, indicating equal benefits of nostalgia therapy across demographic groups.

**Conclusions:**

Nostalgia effectively enhances belongingness and tranquility among older adults. Recalling positive memories provides emotional support, strengthens social connections, and promotes psychosocial stability, making it a valuable intervention in nursing home settings.

**Disclosure of Interest:**

None Declared

